# *pirAB*^*vp*^-Bearing *Vibrio parahaemolyticus* and *Vibrio campbellii* Pathogens Isolated from the Same AHPND-Affected Pond Possess Highly Similar Pathogenic Plasmids

**DOI:** 10.3389/fmicb.2017.01859

**Published:** 2017-10-05

**Authors:** Xuan Dong, Dexi Bi, Hailiang Wang, Peizhuo Zou, Guosi Xie, Xiaoyuan Wan, Qian Yang, Yanping Zhu, Mengmeng Chen, Chengcheng Guo, Zhen Liu, Wenchao Wang, Jie Huang

**Affiliations:** ^1^Laboratory for Marine Fisheries Science and Food Production Processes, Qingdao National Laboratory for Marine Science and Technology, Key Laboratory of Maricultural Organism Disease Control, Ministry of Agriculture, Qingdao Key Laboratory of Mariculture Epidemiology and Biosecurity, Yellow Sea Fisheries Research Institute, Chinese Academy of Fishery Sciences, Qingdao, China; ^2^Department of Pathology, Shanghai Tenth People's Hospital, Tongji University School of Medicine, Shanghai, China; ^3^College of fisheries and life science, Shanghai Ocean University, Shanghai, China; ^4^Shanghai Majorbio Bio-pharm Biotechnology, Shanghai, China

**Keywords:** acute hepatopancreatic necrosis disease, *Vibrio parahaemolyticus*, *Vibrio campbellii*, plasmid, comparative genomics

## Abstract

Acute hepatopancreatic necrosis disease (AHPND) is a severe shrimp disease originally shown to be caused by virulent strains of *Vibrio parahaemolyticus* (VP_AHPND_). Rare cases of AHPND caused by *Vibrio* species other than *V. parahaemolyticus* were reported. We compared an AHPND-causing *V. campbellii* (VC_AHPND_) and a VP_AHPND_ isolate from the same AHPND-affected pond. Both strains are positive for the virulence genes *pirAB*^*vp*^. Immersion challenge test with *Litopenaeus vannamei* indicated the two strains possessed similar pathogenicity. Complete genome comparison showed that the *pirAB*^*vp*^-bearing plasmids in the two strains were highly homologous, and they both shared high homologies with plasmid pVA1, the reported *pirAB*^*vp*^-bearing plasmid. Conjugation and DNA-uptake genes were found on the pVA1-type plasmids and the host chromosomes, respectively, which may facilitate the dissemination of *pirAB*^*vp*^. Novel variations likely driven by IS*Val1* in the genetic contexts of the *pirAB*^*vp*^ genes were found in the two strains. Moreover, the VC_AHPND_ isolate additionally contains multiple antibiotic resistance genes, which may bring difficulties to control its future outbreak. The dissemination of the *pirAB*^*vp*^ in non-*parahaemolyticus Vibrio* also rises the concern of missing detection in industrial settings since the isolation method currently used mainly targeting *V. parahaemolyticus*. This study provides timely information for better understanding of the causes of AHPND and molecular epidemiology of *pirAB*^*vp*^ and also appeals for precautions to encounter the dissemination of the hazardous genes.

## Introduction

Acute hepatopancreatic necrosis disease (AHPND) is a severe shrimp disease that has emerged and been causing heavy losses to the global shrimp farming industry since 2010 (Zhang et al., 2012; Tran et al., 2013; Lee et al., 2015). Outbreaks of AHPND have been reported in recent years in Asia (such as China, Vietnam, Malaysia, Philippines, Thailand) and Latin America (such as Mexico) (Gomez-Gil et al., 2014; Gomez-Jimenez et al., 2014; Kondo et al., 2014, 2015; Nunan et al., 2014; Yang et al., 2014; de la Pena et al., 2015; Lee et al., 2015; Soto-Rodriguez et al., 2015; Chonsin et al., 2016; Restrepo et al., 2016; Han et al., 2017). The disease has caused dramatic drops of shrimp production in affected areas, leading to an estimated loss of over $1 billion per year to the global shrimp farming industry (FAO, 2013). Previous studies have revealed that AHPND has a bacterial etiology (Zhang et al., 2012; Tran et al., 2013; Lee et al., 2015). Earlier researches revealed that it is caused by certain virulent strains of *Vibrio parahaemolyticus*, namely AHPND-causing *V. parahaemolyticus* (VP_AHPND_) (Zhang et al., 2012; Tran et al., 2013; Lee et al., 2015). Recently, it was reported that the bacterial etiology of AHPND also includes *harveyi*-like *Vibrio* (Kondo et al., 2015), *V. owensii* (Liu et al., 2015; Xiao et al., 2017) and *V. campbellii* (Dong et al., 2017a; Han et al., 2017).

*V. parahaemolyticus* is a Gram-negative bacterium that widely inhabits the marine and estuarine environments and it can cause disease in human and animals (Makino et al., 2003; Thompson et al., 2004). A recent study has demonstrated that VP_AHPND_ harbors a plasmid that expresses a deadly toxin Pir^*vp*^ (constituted by PirA^*vp*^ and PirB^*vp*^), which is homologous to the *Photorhabdus* insect-related (Pir) binary toxin (Lee et al., 2015). The Pir^*vp*^ toxin was firstly characterized in the VP_AHPND_ strain 3HP and was produced by plasmid pVA1 (Lee et al., 2015). The Pir^*vp*^ toxin is encoded by the *pirA*^*vp*^ and *pirB*^*vp*^ genes that are located within a fragment delimited by two identical inversely-oriented insertion sequences (IS) IS*Val1*. It is believed the two IS*Val1* with the region in between form a composite transposon called Tn*6264* (Han et al., 2017) or *pirAB*-Tn*903* (Xiao et al., 2017). Interestingly, missing of the fragment was seen on a plasmid sharing homology with pVA1 from a non-AHPND-causing strain M2-36, suggesting that a natural deletion or insertion of *pirAB*^*vp*^ might have occurred (Lee et al., 2015). Other variants with partial deletion were also found (Han et al., 2017). The plasmid pVA1 also carries a cluster of genes related to conjugative transfer (Lee et al., 2015); hence, this plasmid may potentially be able to transfer not only among *V. parahaemolyticus* strains but also to different bacterial species. Indeed, the existence of *pirAB*^*vp*^ has been reported in *harveyi*-like *Vibrio* (one isolate from Vietnam) (Kondo et al., 2015), *V. owensii* (one isolate from China) (Liu et al., 2015; Xiao et al., 2017) and *V. campbellii* (one isolate from China and four from Latin America) (Dong et al., 2017a; Han et al., 2017). We have previously reported the first AHPND-causing *V. campbellii* (VC_AHPND_) strain, 20130629003S01, isolated from Guangxi, China, which contains the *pirAB*^*vp*^ and showed pathogenicity in shrimp (Dong et al., 2017a).

In order to better understand of the spread of *pirAB*^*vp*^, in this study, we compared the pathogenicity and genomic features of the previously reported VC_AHPND_ with that of an isolate of VP_AHPND_ from the same batch of shrimp with AHPND in the same pond. Both strains were *pirAB*^*vp*^-positive and had a similar pathogenic capacity. We further compared their complete genomes and found that the sequences of the *pirAB*^*vp*^-bearing plasmids of both strains were highly homologous, and they shared high homologies with the sequence of pVA1. It suggests that plasmid-mediated interspecies transfer of the hazard genes might have occurred.

## Materials and methods

### Sample collection, bacteria isolation and identification

In June of 2013, samples were collected from AHPND-suspected shrimp farms in Guangxi, China. Hepatopancreas (HP) from diseased shrimp were aseptically disaggregated and streaked on thiosulfate citrate bile salts sucrose (TCBS) plates at 28°C for 12 h. After pure cultures were obtained, a partial 16S rRNA region was amplified with primers 16S_27F and 16S_1492R (Lane, 1991) (Table S1) and sequenced. Partial *rpoD, rctB*, and *toxR* genes were amplified and sequenced as described by Pascual et al. (2010) (Table S1). The concatenated sequences of 16S rRNA, *rpoD, rctB*, and *toxR* loci were aligned. Then the phylogenetic tree was constructed using neighbor-joining analysis with maximum composite likelihood model in MEGA 5 (Tempe, AZ, USA) with 1,000 bootstrap replications. The nucleotide sequences from strain *Vp* 2S01 have been submitted to the GenBank database under accession numbers MF621565 (16S rRNA), MF621566 (toxR), MF621567 (rpoD) and MF621568 (rctB). 16S rRNA, *rpoD, rctB* and *toxR* allele sequences of *Vc* 3S01 were described by our previously report (Dong et al., 2017a). All 16S rRNA, *rpoD, rctB*, and *toxR* allele sequences of reference strains were described by Pascual et al. (2010).

### Detection of *pirA^*vp*^* and *pirB^*vp*^* in the VP_AHPND_ isolate

Bacterial isolates *Vp* 2S01 and *Vc* 3S01 were cultured overnight in Tryptic soy broth with 2% NaCl (TSB+) at 28°C. One milliliter (mL) of the broth culture was boiled for 10 min at 95°C, and the supernatant was obtained by centrifugation, diluted 10-fold with distilled water and used as the template for PCR. PCR was performed using primers VpPirA and VpPirB (Han et al., 2015) (Table S1) as previously described. Protein products were examined by SDS-PAGE and mass spectrometry as previously described (Laemmli, 1970; Wang et al., 2011).

### Ethics statement

Since the Ethical Principles and Guidelines for the Use of Animals of the National Research Council of China applies to vertebrates only, there is no official standard for invertebrates, we adapted its principles to shrimp.

### *Litopenaeus vannamei* challenge test

Before the challenge test, ~1 g healthy white shrimp (*L. vannamei*) were acclimated in the laboratory for 3 days in 50 L seawater at salinity 30 with constant aeration in plastic tanks (density 20 shrimp/tank) at 27 ± 2°C. For the immersion challenge, 20130629002S01 and 20130629003S01 were cultured in TSB+ at 28°C until the OD_600_ reached 0.8–0.9 (approximately 8–12 h). Immersion challenge was performed following the immersion bioassay protocol described by Tran et al. (2013). Simply, the bacterial suspension was adjusted to 1 × 10^8^ cfu·mL^−1^ (with TSB+), and 20 shrimp in each group were immersed in 4 L of this suspension for 15 min. The shrimp and bacterial suspension were then poured into tanks containing 50 L of seawater to give a final bacterial density of 1 × 10^6^ cfu·mL^−1^. The control shrimp were immersed in TSB+ medium. The mortality of each group was recorded every 6 h. Moribund shrimp were fixed with Davidson's alcohol-formalin-acetic acid (DAFA) fixative for histopathological examination. All experiments were done in triplicate.

### Histological confirmation of AHPND

All shrimp sampled for histopathology purposes were fixed with DAFA for 24 h and stained with hematoxylin and eosin (H&E) using routine histological methods described by Lightner (1996). The histological sections were analyzed and photographed by a light microscopy system.

### Sequencing, assembly and annotation of the genome of the VP_AHPND_ isolate

Genomic DNA isolation, sequencing, assembly and annotation were performed as previously described (Dong et al., 2017b). Antibiotic resistance genes were searched against the ResFinder (Zankari et al., 2012). DNA-uptake genes were identified by Blast against the counterparts reported in *Vibrio cholera* (Seitz and Blokesch, 2013).

### The average nucleotide identity (ANI) of genome sequences of VP_AHPND_ isolates

The complete reference genome sequences of *V. parahaemolyticus* strains which downloaded from NCBI were used for comparative genome analysis. The reference *V. parahaemolyticus* strains were M0605 (JALL00000000), D4 (MYFH00000000), FIM-S1708+ (JPLV00000000), NCKU_CV_CHN (JPKU00000000), TUMSAT_DE1_S1 (BAVF00000000), TUMSAT_DE2_S2 (BAVG00000000), NCKU_TV_3HP (JPKS00000000), NCKU_TV_5HP (JPKT00000000), TUMSAT_D06_S3 (BAVH00000000), 1,335 (MYFF00000000), 12297B (MYFG00000000) and A3 (JOKE00000000). ANI value (Richter and Rossello-Mora, 2009) among genomes of *Vp* 2S01 and reference strains was calculated using the JSpecies program. All strains were cut into fragments of 1,020 bp for calculating the ANI values by using the BLAST algorithm (Goris et al., 2007). Next, a distance dendrogram was constructed using the R program.

### Mauve-multiple sequence alignment of *pirAB^*vp*^*-bearing plasmids

The complete sequences of *pirAB*^*vp*^-bearing plasmids downloaded from NCBI were subject to multiple alignment with pVPGX1 and pVCGX1. Those plasmids were pLA16-2 (accession no. CP021148) harbored by VC_AHPND_ strain LA16-V1, pVHvo (accession no. KX268305) harbored by VO_AHPND_ strain SH14, pVPA3-1 (accession no. KM067908) harbored by VP_AHPND_ strain 13-028A3, pVA1 (accession no. KP324996) harbored by VP_AHPND_ strain 3HP, pVPE61a (accession no. AP014860) harbored by VP_AHPND_ strain VPE61, pV110 (accession no. KY498540) harbored by VP_AHPND_ strain v110. Multiple plasmid sequence alignment was performed using Mauve (Darling et al., 2004).

### Antibiotic susceptibility test

Antibiotic susceptibility was determined by the disk diffusion test as described elsewhere (Roque et al., 2001). Briefly, strain suspensions (0.5 MacFarland) of 20130629002S01 and 20130629003S01 were inoculated by lawn onto marine agar and the antimicrobial sensitivity discs positioned. For the present study, antibiotics tested were ampicillin (AMP, 10 μg), aztreonam (ATM, 30 μg), bacitracin (BAC, 0.04 U), cefazolin (CFZ, 30 μg), ceftazidime (CAZ, 30 μg), ceftriaxone (CRO, 30 μg), cephalexin (LEX, 30 μg), cephradine (Rad, 30 μg), ciprofloxacin (CIP, 5 μg), florfenicol (FLO, 30 μg), imipenem (TPM, 10 μg), nitrofurantoin (NIT, 300 μg), norfloxacin (NOR, 10 μg), penicillin (PEN, 10 U), streptomycin (EST, 10 μg), sulfamethoxazole (SMZ, 300 μg), sulfazotrim (SUT, 25 μg), and tetracycline (TCY, 30 μg). The plates were incubated for 24 h at 37°C and the inhibition halos were measured (mm) with vernier caliper. Breakpoints were defined by the guideline (M45-A2, 2010) of Clinical and Laboratory Standards Institute (CLSI). Breakpoints of the antibiotics not listed in the guideline were defined as described by Zhang et al. (2012).

### Statistics

All statistical analyses were performed with SPSS, version 17.0 (SPSS Inc., Chicago, IL, USA). Cumulative mortality was compared by using One-Way ANOVA test. A *P*-value less than 0.05 was considered statistically significant.

### Nucleotide sequence accession numbers

Complete genome sequences of 20130629002S01 (*V. parahaemolyticus*) have been deposited in GenBank under the accession CP020034-CP020037.

## Results

### Two distinct *pirAB^*vp*^*-positive strains isolated from an AHPND epidemic pond displayed similar pathogenicity

**Figure 1:**
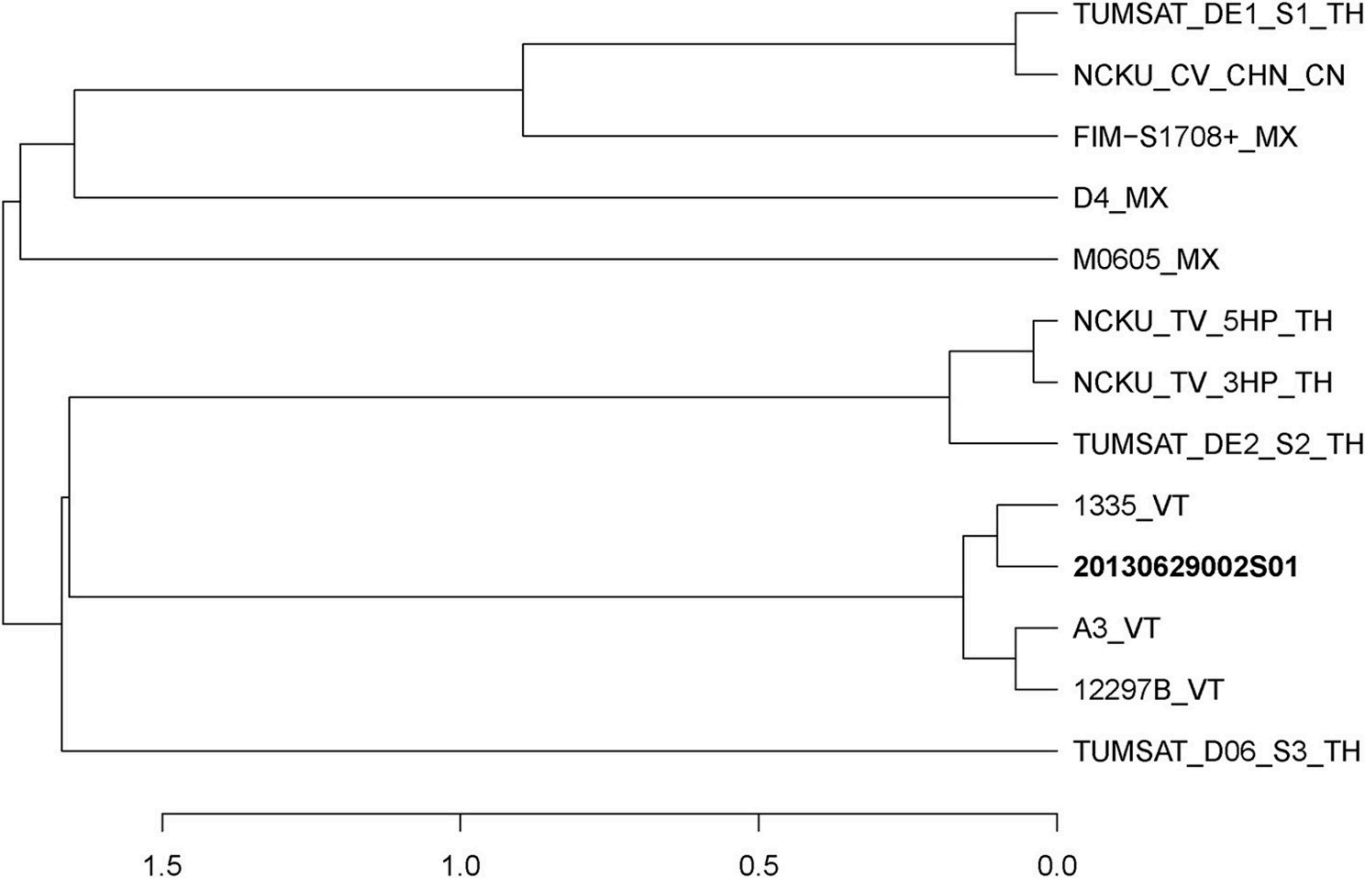
Distance dendrogram among *Vibrio parahaemolyticus* strains based on ANI values. The ANI values were calculated using 13 strains, and all values between every two strains were greater than 98%. The complete genome sequences of *V. parahaemolyticus* (M0605, D4, FIM-S1708+, NCKU_CV_CHN, TUMSAT_DE1_S1, TUMSAT_DE2_S2, NCKU_TV_3HP, NCKU_TV_5HP, TUMSAT_D06_S3, 1335, 12297B and A3) revealed that 20130629002S01 strain has the closest evolutionary relationship (ANI value 99.90%) with an isolate 1,335 isolated from *L. vannamei* in Viet Nam. VT, Vietnam; TH, Thailand; MX, Mexico; CN, China.

In June of 2013, an AHPND-suspected outbreak occurred in a shrimp farm in Guangxi. We have previously reported an AHPND-causing isolate 20130629003S01 (3S01 for short) from the farm, which has been identified as a *pirAB*^*vp*^-positive *V. campbellii* (Dong et al., 2017a). In addition, we isolated another strain, 20130629002S01 (2S01 for short) from the same batch of diseased *Litopenaeus vannamei* in a same pond of the farm. We found strain 2S01 was a *pirAB*^*vp*^-positive *V. parahaemolyticus* (Figure 1). Thus, for short, we used “*Vp* 2S01” for strain 2S01 and “*Vc* 3S01” for strain 3S01 in this paper.

**Figure 2:**
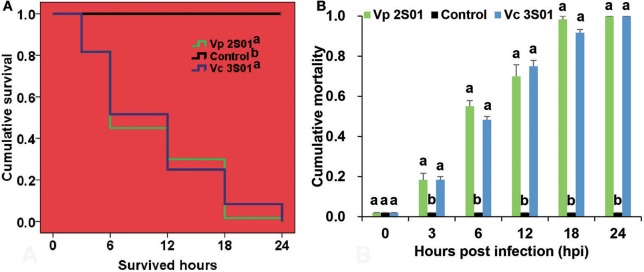
Mortalities induced in the immersion bioassay of *Litopenaeus vannamei*. **(A)** Survival plots of the shrimp in each group. Survival patterns not sharing a common superscript letter following the pattern curve legends were significantly different from each other (*P* < 0.05). **(B)** Mortality of shrimp in each group. Immersion bioassay was performed in triplicate. Error bars indicate standard error of measurement (SEM). Different lowercase letters indicate significant differences (*p* < 0.05).

Shrimp immersed with *Vp* 2S01 or *Vc* 3S01 suspension were observed to develop typical gross signs of AHPND within 6 h. AHPND shrimp from the *Vp* 2S01- and *Vc* 3S01-infected groups had a pale, atrophied HP, and an empty stomach (ST) and midgut (MG), compared to the normal ones that had a normal size HP with dark orange color and a full ST and midgut MG. The survival pattern of different groups was illustrated in Figure 2A. The control group showed no mortality whereas *Vp* 2S01- and *Vc* 3S01-infected groups showed 100% mortality within 24 h. As shown in Figure 2B, *Vp* 2S01 and *Vc* 3S01 had similar pathogenicity (*P* > 0.05). Histopathological examination of moribund shrimp samples from *Vp* 2S01- and *Vc* 3S01-infected shrimp were used to confirm AHPND, which also revealed similar presence of AHPND lesions in HP (Figure S1).

### Genomic feature comparison of *Vp* 2S01 and *Vc* 3S01

The genome of *Vc* 3S01 has been previously sequenced (Dong et al., 2017b). Genome sequences of *Vp* 2S01 were determined here using the PacBio RS II sequencing platforms with 189× coverage. *Vp* 2S01 contains two circular chromosomes (I and II) and two circular plasmids (pVPGX1 and pVPGX2), while *Vc* 3S01 contains two circular chromosomes (I and II) and four plasmids (pVCGX1 to pVCGX4) (Dong et al., 2017b). Plasmids pVPGX1 and pVCGX1 are homologous to *V. paraheamolyticus* plasmids pVA1, pVPA3-1 with overall 99% nucleotide identities and partial pFORC4 (accession no. CP009849) with a 94% identity.

Significantly, genes related to DNA transfer were found in both strains. Conjugative transfer were identified on plasmids pVPGX1, pVCGX1, pVCGX3 and pVCGX4 and the chromosome I of *Vc* 3S01. Plasmids pVPGX1 and pVCGX1 carry *tra*-*trb* genes that are highly similar to the counterparts on pVA1 (Lee et al., 2015). The plasmids pVCGX3, pVCGX4 and the chromosome I of *Vc* 3S01 contain *virB*/*D* clusters (Table S2). The ones on pVCGX3 and the chromosome I are near identical, but distinct to the one on pVCGX4. The *virB*/*D* genes encoding a type IV secretion system were well known on the *Agrobacterium tumefaciens* plasmid Ti as being responsible for effector translocation and DNA conjugation (Shirasu et al., 1990; Christie, 2004). In addition, many other homologous *virB*/*D* clusters were characterized to be self-transmissible modules of conjugative plasmids and integrative and conjugative elements (ICEs) (Bi et al., 2013). Moreover, intact sets of genes related to DNA uptake were identified on the chromosomes I of both strains (Table 1). Their products display 35.7–95.4% amino acid identities to the counterparts of *V. cholerae* O1 biovar El Tor str. N16961, which are also encoded by chromosome I. The DNA-uptake genes in *V. cholerae* N16961 has been proved to be required for efficient natural transformation (Seitz and Blokesch, 2013).

**Table 1:** Genes required for efficient natural transformation of *Vp* 2S01 and *Vc* 3S01.

Reference locus_tag^*^	**Gene_name**	***Vc* 3S01 homologs**	**Vc 3S01_location**	**Vc 3S01 % identity**	**Vp 2S01 homologs**	**Vp 2S01_location**	**Vp 2S01 % identity**
*VC0462*	*pilT*	*Vc3S01_0475*	chromosome I 549635…550675	86.4	*Vp2S01_0459*	chromosome I 507234…508274	87.3
*VC0543*	*recA*	*Vc3S01_0535*	chromosome I 611769…612812	95.1	*Vp2S01_0520*	chromosome I 571980…573023	95.4
*VC0857*	*VC0857*	*Vc3S01_2591*	chromosome I 2927019…2927453	41.8	*Vp2S01_2489*	chromosome I 2679066…2679497	44.5
*VC0858*	*VC0858*	*Vc3S01_2590*	chromosome I 2926915…2926400	37.4	*Vp2S01_2488*	chromosome I 2678932…2678447	37.8
*VC0859*	*VC0859*	*Vc3S01_2589*	chromosome I 2926390…2925764	37.9	*Vp2S01_2487*	chromosome I 2678438…2677812	36.1
*VC0860*	*VC0860*	*Vc3S01_2588*	chromosome I 2925764…2924436	36.4	*Vp2S01_2486*	chromosome I 2677812…2676484	35.7
*VC0861*	*VC0861*	*Vc3S01_2587*	chromosome I 2924446…2924036	62.8	*Vp2S01_2485*	chromosome I 2676494…2676084	63.5
*VC1612*	*VC1612*	*Vc3S01_1463*	chromosome I 1610447…1610088	63.6	*Vp2S01_1405*	chromosome I 1468383…1467658	63.4
*VC1879*	*comEC*	*Vc3S01_2263*	chromosome I 2554872…2552614	41.0	*Vp2S01_2164*	chromosome I 2321516…2319258	41.0
*VC1917*	*ComEA*	*Vc3S01_2319*	chromosome I 2617558…2617271	58.3	*Vp2S01_2235*	chromosome I 2406333…2406040	64.1
*VC2423*	*pilA*	*Vc3S01_0564*	chromosome I 643331…642915	36.2	*Vp2S01_0548*	chromosome I 603064…602636	51.3
*VC2424*	*pilB*	*Vc3S01_0563*	chromosome I 642915…641230	73.7	*Vp2S01_0547*	chromosome I 602636…600951	74.0
*VC2425*	*pilC*	*Vc3S01_0562*	chromosome I 641195…639972	73.8	*Vp2S01_0546*	chromosome I 600926…599703	74.1
*VC2426*	*pilD*	*Vc3S01_0561*	chromosome I 639899…639030	75.0	*Vp2S01_0545*	chromosome I 599638…598769	73.3
*VC2630*	*pilQ*	*Vc3S01_0320*	chromosome I 365090…366832	77.7	*Vp2S01_0314*	chromosome I 345396…347081	77.7
*VC2631*	*pilP*	*Vc3S01_0319*	chromosome I 364540…365055	56.4	*Vp2S01_0313*	chromosome I 344786…345301	57.6
*VC2632*	*pilO*	*Vc3S01_0318*	chromosome I 363957…364550	72.3	*Vp2S01_0312*	chromosome I 344203…344796	72.6
*VC2633*	*pilN*	*Vc3S01_0317*	chromosome I 363389…363964	60.5	*Vp2S01_0311*	chromosome I 343632…344210	62.4
*VC2634*	*pilM*	*Vc3S01_0316*	chromosome I 362389…363405	49.4	*Vp2S01_0310*	chromosome I 342632…343648	48.4
*VC2719*	*comF*	*Vc3S01_3047*	chromosome I 3434182…3434907	46.6	*Vp2S01_2993*	chromosome I 3221591…3222316	47.9

* *Reference VC numbers were described by Seitz and Blokesch (2013)*.

Antibiotic resistance genes were found in both strains, which will be described later. Neither *Vp* 2S01 nor *Vc* 3S01 contain genes (*scrB*) that encode the sucrose hydrolase. Both *Vp* 2S01 and *Vc* 3S01 formed green and round colonies on TCBS plate. However, genes encoding the sucrose hydrolase were found in the genome sequence of AHPND-causing strain SH14 of *V. owensii* (Liu et al., 2015).

### Complete plasmid sequences comparison of *pirAB^*vp*^*-bearing plasmids

**Figure 3:**
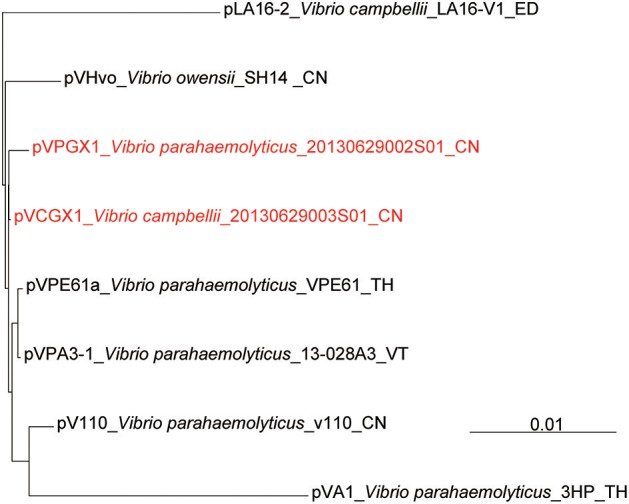
Phylogenetic reconstruction based on complete sequences of *pirAB*^*vp*^-bearing plasmids. The complete reference sequences of *pirAB*^*vp*^-bearing plasmids (pLA16-2, pVHvo, pVPA3-1, pVA1, pVPE61a, pV110) were downloaded from NCBI. Multiple plasmid sequences alignment among *pirAB*^*vp*^-bearing plasmids from *Vp* 2S01, *Vc* 3S01 and reference strains was calculated using mauve (Darling et al., 2004). VT, Vietnam; TH, Thailand; ED, Ecuador; Mexico; CN, China.

**Figure 4:**
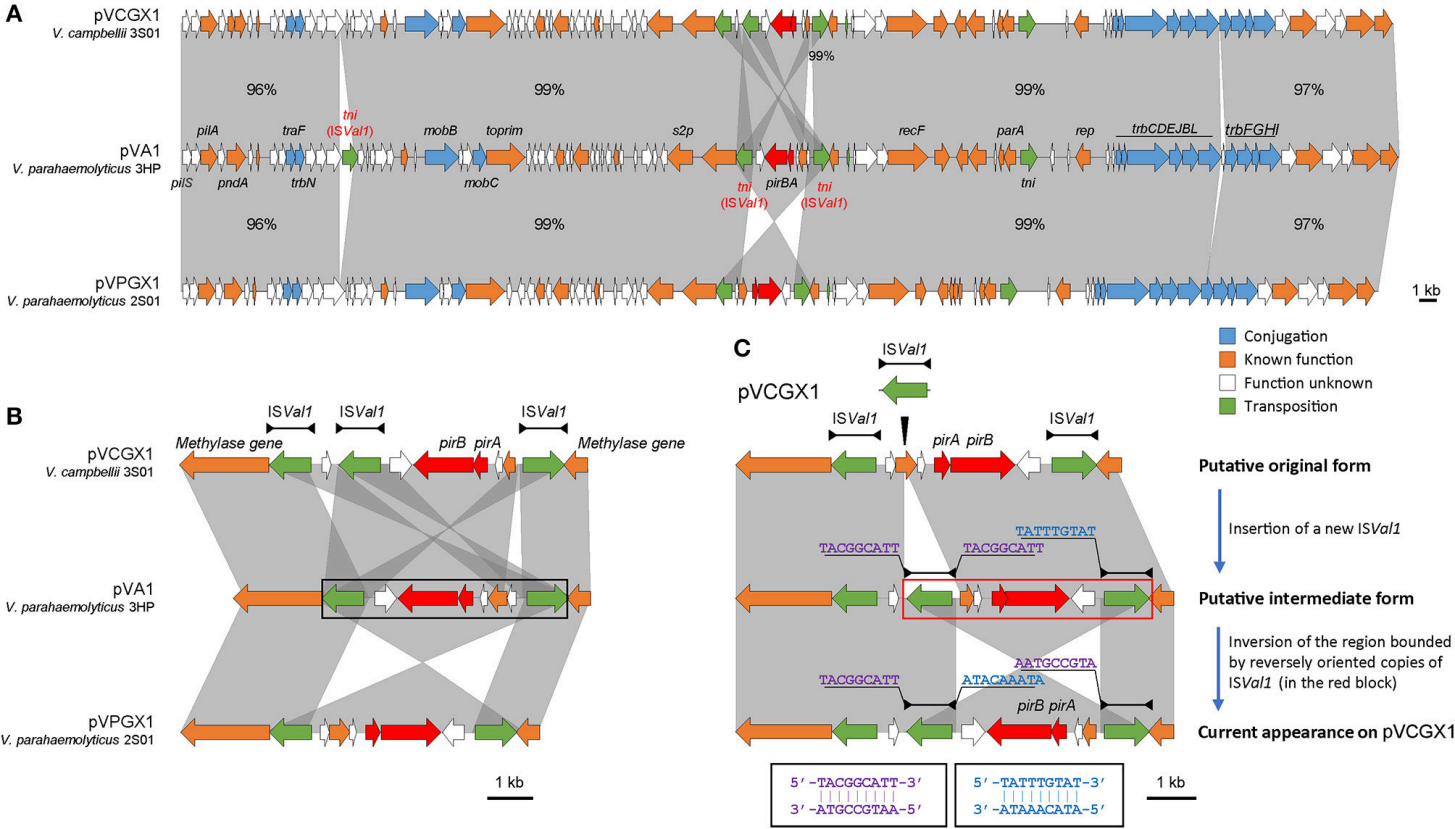
Analysis of *pirAB*^*vp*^-bearing plasmids pVCGX1 and pVPGX1. **(A)** Comparison of plasmids pVCGX1 and pVPGX1 with pVA1. Percentages indicate the identities in nucleotide level between syntenic regions. Percentages are not given for 100% identical regions. **(B)** A zoom-in view of the genetic context of *pirAB*^*vp*^. Horizontal lines shown above the schematics, with both ends demarcated by solid triangles to indicated IRs, represent the IS*Val1*. Black block denotes the 5.5-kb *pirAB*^*vp*^ fragment describe in the text. **(C)** Putative evolution process of the genetic context of *pirAB*^*vp*^ on plasmid pVCGX1. Thin triangle indicates IS insertion. In the red block is the putative region going inversion. Nucleotide sequences flanking the IS*Val1* elements are shown in 5′ → 3′. Sequences in identical colors are reverse-complement to each other as denoted in the black blocks. Gene organization is drawn to scale. Red arrows indicate *pirAB*^*vp*^ genes. Genes with other known functions are denoted using different colors. Syntenic regions were highlighted in gray.

The *pirAB*^*vp*^ genes located on plasmids pVPGX1 in *Vp* 2S01 and pVCGX1 in *Vc* 3S01. The pVPGX1 and pVCGX1 displayed a 99% nucleotide identity spanning the entire sequences, while each of the two plasmids displayed a 96–100% nucleotide identity to pVA1 (**Figure 4A**). Further comparative analysis found that the two plasmids shared high homologies with other *pirAB*^*vp*^-bearing plasmids from AHPND-causing *Vibrio* strains (Figure S2). Importantly, pVPGX1 and pVCGX1 shared the closest relationship among all the compared plasmids from AHPND-causing *Vibrio* (Figure 3). More interestingly, different versions of the genetic contexts of *pirAB*^*vp*^ (*pirAB*^*vp*^ contexts) were observed in all plasmids when compared (Figure S2).

### Sequence analysis of the *pirAB^*vp*^* contexts of plasmids pVPGX1 and pVCGX1

In pVA1, the *pirAB*^*vp*^ genes locate within a 5.5-kb fragment ended with two inversely oriented copies of IS*Val1* which belonged to the IS*903* group of the IS*5* family (length includes the two IS elements). The *pirAB*^*vp*^ were located among some small ORFs between the two IS*Val1*. IS*Val1* was 1,054 bp in length and demarcated by 18-bp perfect inverted repeats (5′-GGCTTTGTTGCGTAATTC-3′). It displayed a 92% nucleotide identity to IS*Va2*, which was initially found in *V. anguillarum* (Tolmasky and Crosa, 1995). In pVPGX1, an inversion of the 5.5-kb *pirAB*^*vp*^ fragment was seen. Transformation of two configurations was likely due to recombination between two opposite IS*Val1* (Partridge, 2011). In contrast, the *pirAB*^*vp*^ contexts in pVCGX1 had a more complex structure. The *pirAB*^*vp*^ fragment had an extra copy of IS*Val1*, which divided the fragment into two parts. The left part was inversely syntenic to pVA1, while the right part was directly syntenic to pVA1 (Figure 4B). In addition, an IS*Val1* located downstream of *trbN* and a 217-bp fragment within the *trb* gene cluster in pVA1 were absent in pVPGX1 and pVCGX1 (Figure 4A).

We also analyzed the *pirAB*^*vp*^ contexts in 12 other sequenced AHPND-causing *Vibrio* genomes (Table S3). Novel variant was not detected using the current data except that the *pirAB*^*vp*^ fragment in *V. parahaemolyticus* M0605 likely located in a different site of a pVA1-type plasmid among them. The site was close to the insertion site of the solitary IS*Val1* in pVA1. Note that since most of the reported genomes were unfinished, there is a possibility that there are undetected novel variants lying in the undetermined gaps.

### Antibiotic resistance

Antibiotic resistance of *Vp* 2S01 and *Vc* 3S01 were detected. The resistance gene profiles of the two strains were also revealed. *Vp* 2S01 harbored two antibiotic resistance genes *tet(35)* and *tet(34)* on chromosome I, while *Vc* 3S01 contains *tet(35)* on chromosome I and *sul2, strAB, tet(A), strB-2* (extra copy) and *floR* on pVCGX2. The results of disk diffusion test showed that *Vp* 2S01 was only susceptible to florfenicol. It was intermediate to ceftriaxone, nitrofurantoin, norfloxacin. Significantly, *Vc* 3S01 was resistant to a wider spectrum of antibiotics as being resistant or intermediate to all test antibiotics (Table S4).

## Discussion

We compared a VP_AHPND_ and a VC_AHPND_ isolate from the same AHPND-affected pond. They displayed a similar pathogenicity and both contained the *pirAB*^*vp*^ genes carried by plasmids that are highly homologous to pVA1. This study reveals the dissemination of the hazardous genes in *V. campbellii*, which is likely due to interspecies horizontal gene transfer.

Although VP_AHPND_ is almost the only pathogen known to cause AHPND, studies have indicate that non-*parahaemolyticus* AHPND-causing *Vibrio* is emerging. Indeed, certain *V. harveyi*-like, *V. owensii*, and *V. campbellii* strains have been detected to cause AHPND and to be *pirAB*^*vp*^-positive (Kondo et al., 2015; Liu et al., 2015; Dong et al., 2017a; Han et al., 2017; Xiao et al., 2017). However, only a limited number of the isolates were characterized and compared. The VC_AHPND_ we previously isolated displays a similar level of pathogenicity to VP_AHPND_. Immersion challenge for *L. vannamei* shrimp demonstrates that *Vc* 3S01-infected shrimp present similar mortality, AHPND manifestations and pathology to *Vp* 2S01-infected shrimp. It seems that the pathogenicity encoded by *pirAB*^*vp*^ is independent from host bacteria within closely related *Vibrio* species. Remarkably, the *pirAB*^*vp*^ genes may be not originated from *V. parahaemolyticus*. A recent study has demonstrated that non-virulent *V. parahaemolyticus* becomes VP_AHPND_ via acquiring a plasmid named pVA1 that expresses a deadly toxin Pir^*vp*^ (Lee et al., 2015). Additionally, the spread of the VP_AHPND_ via diseased animals and contaminated water accelerates the pandemic of this disease. Similarly, other *Vibrio* species also could become pathogenic by acquisition of this plasmid. The prevalence of VP_AHPND_ may suggest either they have better capacity of colonization or they are the first pathogenic host bacterium of this plasmid.

The *pirAB*^*vp*^-bearing plasmids pVPGX1 and pVCGX1 are highly homologous to pVA1, suggesting the occurrence of horizontal transfer of the pVA1-type plasmid. The pVA1-type plasmids with or without *pirAB*^*vp*^ genes have been found in many *Vibrio* species (Xiao et al., 2017). Plasmid is an important factor driving the dynamic gene flow and promoting the fitness of host bacteria (Laurenceau et al., 2013; Seitz and Blokesch, 2013; Matthey and Blokesch, 2016). Plasmid can be acquired mainly through transformation or conjugation. It has been reported that the pVA1 plasmid contained a set of conjugative transfer genes, which suggests that pVA1 might be self-transmissible (Lee et al., 2015). Meanwhile, homologs of genes encoding a DNA-uptake machinery reasonable for natural competence of *V. cholera* (Seitz and Blokesch, 2013) were also found in the VP_AHPND_ and VC_AHPND_ isolates. Given that the two isolates were from the same origin and the prevalence of VP_AHPND_, there is a possible scenario that the pVA1-type plasmid pVCGX1 in VC_AHPND_ was originally acquired from VP_AHPND_, via either self-mediated conjugation or uptake of the free plasmids released by dead VP_AHPND_ cells. Interestingly, the *Vc* 3S01 contains 4 heterogeneous plasmids. It's unusual that so many different plasmids exist in one *Vibrio* cells. It may be due to the strain *Vc* 3S01 has a strong capability to obtain other plasmid. However, whether these transfer genes facilitate the intra-species dissemination of *pirAB*^*vp*^ still awaits investigation. Moreover, *Vc* 3S01 is also “armed” with multiple antibiotic resistance genes, which will bring difficulties to control its future spread. Therefore, the transfer of these genes into a new host bacterium not only increases the complexity of causative agents, but also leads to a potential threat with no drug for treatment.

Despite the synteny of the carrier plasmids, the *pirAB*^*vp*^ genes have dynamic contexts. We assumed the configuration showed in Figure 4C was resulted from the insertion of a new IS*Val1* followed by recombination. We subsequently analyzed the flanking sequences of relevant IS*Val1* elements to identify the recombination event using the method proposed by Partridge (2011) and found that the right part might have gone through an inversion event after the insertion of a new IS*Val1*. This right part was bounded by the oppositely oriented middle IS*Val1* and right IS*Val1*. A 9-bp left flank (5′-TACGGCATT-3′) of the middle IS*Val1* was reverse-complement to that of the right IS*Val1* (5′-AATGCCGTA-3′). If we inverse the current form of this region, the 9-bp direct repeats flanking the middle IS*Val1* could be restored and the direction of synteny to pVA1 would be consistent to that of the left part. Thus, an evolution process of *pirAB*^*vp*^ contexts was deduced. A new IS*Val1* inserted into the original *pirAB*^*vp*^ contexts, generating 9-bp direct repeats (5′-TACGGCATT-3′) and a sub-region that was also bounded by two opposite IS*Val1*. Then inversion of the sub-region occurred, resulting in the current form.

The *pirAB*^*vp*^ context initially revealed on pVA1 is bounded by two identical but inversely oriented IS elements, IS*Val1*. Mobile genetic elements such as IS elements, transposons, integrons and genomic islands are important factors shaping genetic contexts of antimicrobial resistance genes and virulence genes (Partridge, 2011; Stokes and Gillings, 2011). Inversely oriented IS elements close to each other are potential targets for homologous recombination (Partridge, 2011). Unsurprisingly, an inversion form of *pirAB*^*vp*^ context was found on pVPGX1. It has been suggested that the natural acquisition or deletion of the *pirAB*^*vp*^ genes is due to transposition or homologous recombination (Lee et al., 2015). However, we prefer the former theory since the recombination results in inversion instead of excision or integration of *pirAB*^*vp*^ contexts. The two IS*Val1* might have formed a composite transposon. Moreover, it seems that IS*Val1* is also a factor that complicates the *pirAB*^*vp*^ context since the more complex configuration on pVCGX1 is very likely due to the insertion of a new IS*Val1* followed by recombination. Therefore, we propose that IS*Val1* plays a role not just in the translocation of the *pirAB*^*vp*^ genes but also in the modulation of their contexts.

TCBS plate is currently used as a simple tool in many shrimp farms for monitoring and forewarning the *V. parahaemolyticus*-associated risk that may cause AHPND, as most *V. parahaemolyticus* strains develop green colonies (Non-sucrose-fermenting bacterial colonies are covered by the green color). This method works for *V. campbellii* as well. Indeed, neither *Vp* 2S01 nor *Vc* 3S01 contains genes that encode the sucrose hydrolase, such as *scrB* gene (Reid and Abratt, 2005). Other *Vibrio* species, like *V. alginoluticus, V. cholerae* carry the sucrose hydrolase genes and develop yellow colonies. A *V. owensii* strain from a shrimp farm suffering AHPND in Haiyang of Shandong Province in 2017 was also tested as *pirAB*^*VP*^ positive. The strain was tested on TCBS plate and developed a yellow colony (unpublished results). Therefore, there is a potential risk of negative detection of pathogenic strains when using the TCBS plate method given the increasing intra-species dissemination of the *pirAB*^*VP*^ genes. We here suggest both green and yellow colonies should be tested under such circumstance.

AHPND has caused severe production collapses and heavy economic losses in Asia and Latin America. (Zhang et al., 2012; FAO, 2013; De Schryver et al., 2014; Nunan et al., 2014; de la Pena et al., 2015; Lee et al., 2015). Actions to bring the disease to an end are in urgent need. This study demonstrates the dissemination of the *pirAB*^*vp*^ genes in *V. campbellii* and thus sheds light on the molecular epidemiology of the virulence genes. Moreover, acquisition of the pVA1-type, *pirAB*^*vp*^-bearing plasmids in diverse *Vibrio* species increases the complexity of causative agents of AHPND and their potential threat to the shrimp industry. Therefore, our study provides timely information for better understanding of the causes, epidemiological features of AHPND, as well as developments of response measures and prevention and control strategies.

## Author contributions

XD and DB designed and conducted the study, performed most of the experiments, and as well as HW wrote the manuscript. PZ, QY, XW, MC, CG, and WW performed the biological experiments. ZL assembled preliminary sequences and analysis. HW, QY, GX, XW, YZ, and QW discussed the results and modified the manuscript. JH designed the study and wrote the manuscript. All authors reviewed the manuscript.

### Conflict of interest statement

The authors declare that the research was conducted in the absence of any commercial or financial relationships that could be construed as a potential conflict of interest.
