# Supplementary material for: pirABvp-Bearing Vibrio parahaemolyticus and Vibrio campbellii Pathogens Isolated from the Same AHPND-Affected Pond Possess Highly Similar Pathogenic Plasmids
Source: Front Microbiol. 2017 Oct 5;8:1859. doi: 10.3389/fmicb.2017.01859 (PMC5633605; doi:10.3389/fmicb.2017.01859)
Supplement: Supplementary file 2 [file Image1.pdf]

PirAB^{vp}-bearing *Vibrio parahaemolyticus* and *Vibrio campbellii* pathogens isolated from the same AHPND-affected pond possess highly similar pathogenic plasmids

Authors and affiliations:

Xuan Dong[1†], Dexi Bi[2†], Hailiang Wang[1], Peizhuo Zou[1, 3], Guosi Xie[1], Xiaoyuan Wan[1], Qian Yang[1], Yanping Zhu[1], Mengmeng Chen[1, 3], Chengcheng Guo[1], Zhen Liu[4], Wenchao Wang[1], Jie Huang[1*]

[1] Laboratory for Marine Fisheries Science and Food Production Processes, Qingdao National Laboratory for Marine Science and Technology; Key Laboratory of Maricultural Organism Disease Control, Ministry of Agriculture; Qingdao Key Laboratory of Mariculture Epidemiology and Biosecurity; Yellow Sea Fisheries Research Institute, Chinese Academy of Fishery Sciences, Qingdao, China
[2] Department of Pathology, Shanghai Tenth People's Hospital, Tongji University School of Medicine, Shanghai, China
[3] College of fisheries and life science, Shanghai Ocean University, Shanghai, China
[4] Shanghai Majorbio Bio-pharm Biotechnology, Shanghai, China
***Corresponding author.** Tel.: +86-0532-85823062; Fax: +86-0532-85811514; E-mail: huangjie@ysfri.ac.cn (Jie Huang).
[§] These authors contributed equally to this work.

Xuan Dong: dongxuan311@163.com
Dexi Bi: bidexi2@163.com
Hailiang Wang: whl846130@126.com
Peizhuo Zou: zoupeizhuo@qq.com
Guosi Xie: xiegs@ysfri.ac.cn
Xiaoyuan Wan: wanxy@ysfri.ac.cn
Qian Yang: yangqianouc@163.com
Yanping Zhu: yanpingzhu1983@163.com
Mengmeng Chen: mmchen0104@163.com
Chengcheng Guo: 977560319@qq.com
Zhen Liu: zhen.liu@majorbio.com
Wenchao Wang: 903899839@qq.com
Jie Huang: huangjie@ysfri.ac.cn

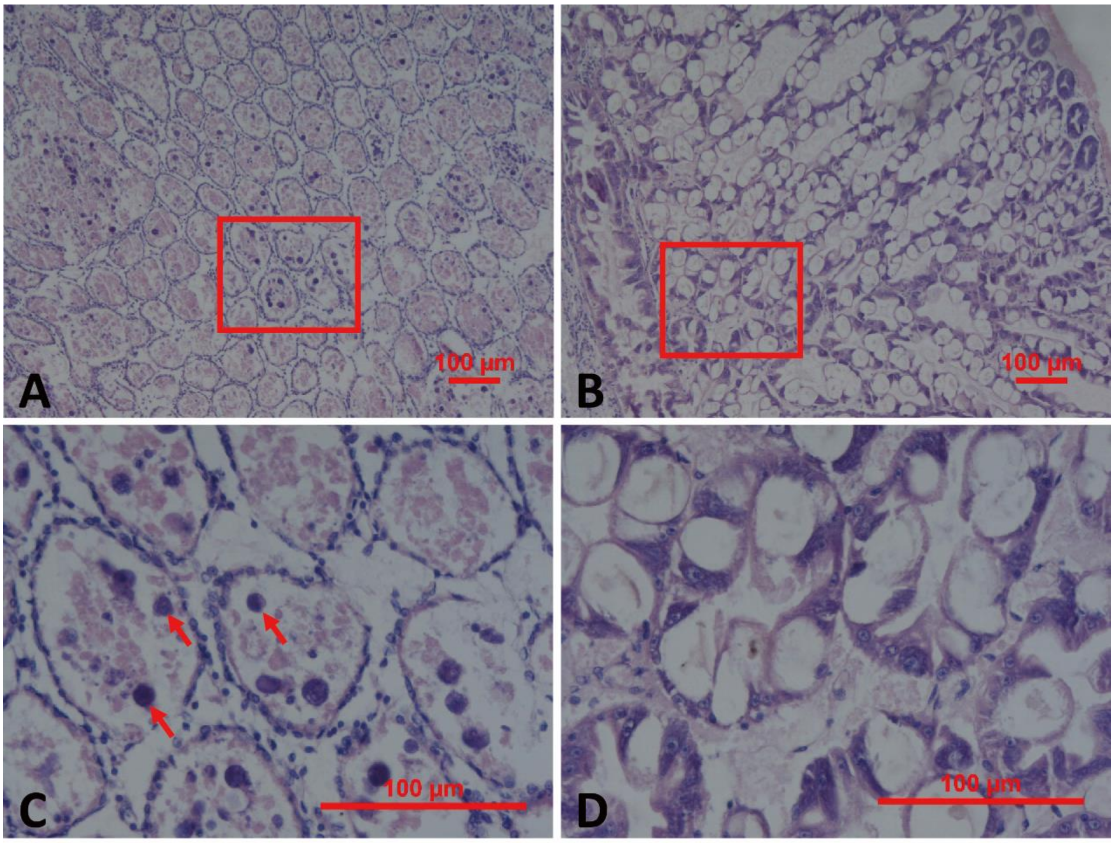

Supplementary Figure S1. H&E stained histological sections of the hepatopancreas of *Litopenaeus vannamei* from the challenge tests. A-B, 100× view field. C-D, Zoom-in view (400×) of the corresponding areas in the red blocks in A-B. A and C: *Vp* 2S01-infected group; B and D, Negative-control group; A and C: AHPND pathology characterized by sloughing of hepatopancreatic tubule epithelial cells (arrows); B and D: Normal shrimp hepatopancreatic histology. Scale bars = 100 μm.

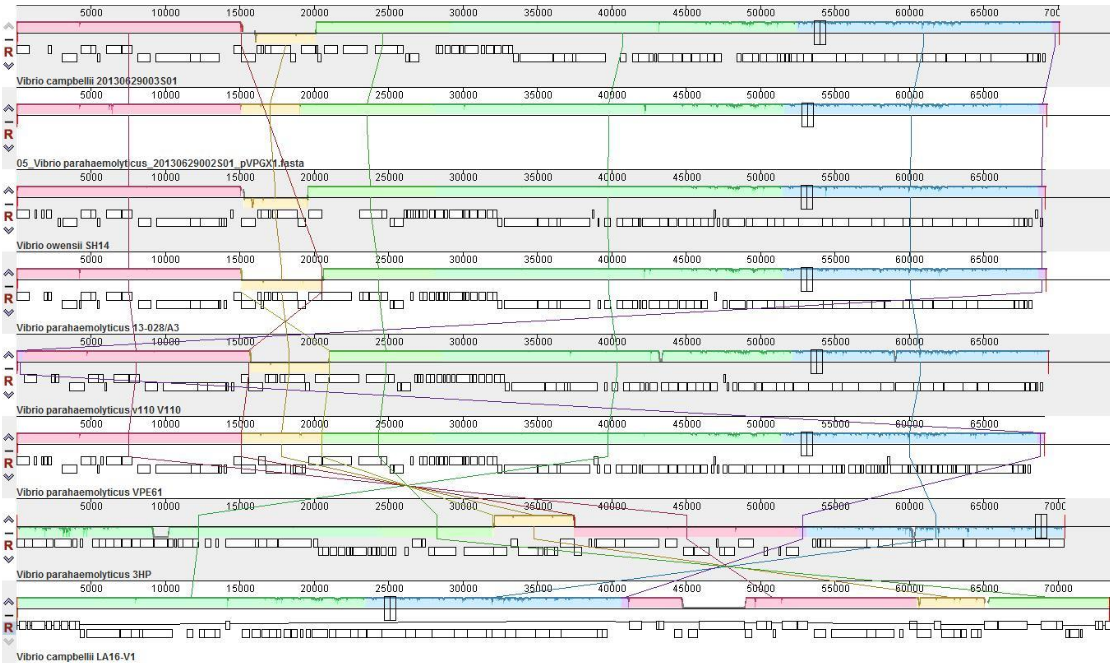

Supplementary Figure S2. Complete sequences comparison of *pirAB^{vp}*-bearing plasmids.

The complete reference sequences of *pirAB^{vp}*-bearing plasmids (pLA16-2, pVHvo, pVPA3-1, pVA1, pVPE61a, pV110) were downloaded from NCBI. Multiple plasmid sequences alignment among *pirAB^{vp}*-bearing plasmids from *Vp* 2S01, *Vc* 3S01 and reference strains was calculated using mauve (Darling et al., 2004).

Colored blocks in the first genome are connected by lines to similarly colored blocks in other genomes. These lines indicate which regions in each genome are homologous.

Darling, A.C., Mau, B., Blattner, F.R., and Perna, N.T. (2004). Mauve: multiple alignment of conserved genomic sequence with rearrangements. *Genome Res* 14(7), 1394-1403. doi: 10.1101/gr.2289704.
